# Unpacking polycontextural complications: a reflexive systems-theoretical heuristic for tracing intersystemic implementation processes in education for sustainable development

**DOI:** 10.3389/fsoc.2026.1883119

**Published:** 2026-08-26

**Authors:** Simon Schmalen, Nils Mühlenbrock

**Affiliations:** Institute of Sociology, RWTH Aachen University, Aachen, Germany

**Keywords:** systems theory, organizational translation, polycontexturality, Luhmann, systems level differentiation, theorizing, sociology of transformation, education for sustainable development

## Abstract

This paper outlines meta-theoretical and research-practical ways of engaging with polycontextural constellations in intersystemic organizational implementation endeavors. Through the case of the implementation of Education for Sustainable Development (ESD) in primary schools, we develop a systems-theoretical heuristic for empirically analyzing intersystemic dynamics while explicitly systematizing the theory-building process. Drawing on qualitative interviews and ethnographic data from three school development processes in Germany, we reconstruct organizational re-combination processes from a differentiation-theoretical perspective. Rather than applying the theory subsumptively, we employ social-ontological and society-theoretical concepts in a reflexive oscillation between empirical and theoretical enrichment. Along the horizontal and vertical axes of Luhmannian system(-level) differentiation, we systematize translation cascades and identify two polycontextural complications that only become analytically accessible through systematic analysis of code dynamics and operational couplings. At the first-order level, “contingency incongruence” emerges as competency-based learning concepts encounter a binary-coded contingency of the established program. Interorganizational workshops convened between schools and consultancing organizations to address this reveal a second-order complication: The “a-addressability” of workshop participants renders the intervention unreachable beyond the boundaries of the emergent third system. The generalization of these findings suggests that polycontextural complications are not incidental but structurally inherent to intersystemic implementation processes.

## 1 Introduction

Addressing the existential problems of climate change falls largely to organizations, the central coordinating units of the *society of organizations* that characterizes modern social life (Perrow, 1992; Arnold et al., 2025). They cast problems—and the societal expectations that follow from them—into concrete, decidable forms that can be circulated and delegated across organizational boundaries, and that are thereby meant to enable the coordination and cooperation necessary to address existential problems (Besio and Meyer, 2020; Besio and Tacke, 2024; Lutz and Mölders, 2025). Sustainability, for instance, is given programmatic form in the UN Sustainable Development Goals (SDGs), which do not arise of their own accord but must be decided upon (Howard-Grenville and Spengler, 2022). Yet despite this organizational processing achievement, and despite broad consensus on the existential nature of anthropogenic climate change, large-scale transformation continues to unfold only slowly and remains difficult to implement.

This tension, we argue, is rooted in a more fundamental problem structure that, over the course of the transformation process, manifests as a complexity whose systemic identity makes it difficult to grasp. On the one hand, within the context of the pervasive “polycrisis” (Tooze, 2022; Rakowski et al., 2025), the climate crisis is frequently treated as *unum ex multis* and is thereby accorded comparatively low priority in political responses and corresponding measures. On the other hand, constructive coordination and intentional transformation are impeded by a prior uncertainty and divergence: what, exactly, is the threat to be addressed? Is it rising annual maximum temperatures (Copernicus, 2026), the growing “attraction of submission to an external authority” within formerly democratic societies (Simon, 2024, p. 26), or rising CO₂ prices (OECD, 2025)? While it may seem trivial that each of these framings—those of science, politics, or the economy—captures only one perspective on the same phenomenon, each of these observational standpoints remains oblivious to the blind spots inherent in its own diagnosis. The climate crisis itself—much like societal approaches to sustainable development—thus presents itself as a poly*optical* problem (Kron and Winter, 2021, pp. 72–73). Once processed through the differentiated “global modes of access” (Türk, 1995, p. 173) across (functionally) differentiated societal spheres, it multiplies poly*contexturally*. Polycontexturality thus marks the constitution of an event through various processing systems at the operative level (Luhmann, 1990a, p. 666). The multiplication does not remain confined to the level of societal problem definitions: it unfolds a corresponding complexity at the organizational level as well, where neither the, respectively, specified expectations nor the modes of operation and perspectives of the organizations involved readily converge. If problem definitions already diverge in this way, then there is no single trajectory of sustainable development that could command general assent, “but rather a plurality of developmental claims; not a single sustainability, but a plurality of sustainabilities that differ from system to system” (Fuchs, 2008, pp. 7–8). Sustainability ultimately becomes an “empty formula” (Lutz and Mölders, 2025, p. 69) and organizational coordination, shared strategies, or even steering thus increasingly elude us.

One such organizationally workable expectation is the Education for Sustainable Development (ESD) initiative, whose organizational implementation process in German primary schools serves as the empirical case study of this article. As a central element of the fourth SDG (“Quality Education”), ESD brings education into play as a key lever for addressing societal challenges (UNECE, 2022; BMBFSFJ, 2026). By contrast, educational research stresses the field’s inherent complexity and challenges the universalistic expectations implicit in “educationalizing social problems” (Labaree, 2008; Gherardi, 2021; see also Swennen, 2024). Education is thus caught in an ambivalent position: invoked as a (supposed) solution to “external” problems while simultaneously constituting a problem in its own right (El-Mafaalani, 2021).

This tension materializes at the organizational level: transformative change is addressed to the organization “school” as a politically only vaguely specified societal expectation, which is to be promoted while integrating sustainability issues into everyday school practice. Primary schools occupy a particular position in this regard, making them an especially instructive case for examining such organizational translation processes in education. As the first sustained organizational point of access to future members of society, they are assigned a key role in addressing and shaping individuals with respect to broader societal solutions (Lischka-Schmidt, 2023; Lange et al., 2026). Yet the organizational implementation of initiatives like ESD here proves structurally fraught, marked by considerable (structural) hurdles and implementation deficits (Timm and Barth, 2021; Budde and Blasse, 2023; Hung and Pan, 2025). Primary schools engaging with ESD are consequently confronted with a growing complexity of problems in which not only the crises themselves, but also their entanglements (poly*crisis*), are constituted and multiplied (poly*contexturality*) through a multiplicity of problematizations and demands for solutions articulated from incommensurable observational standpoints (poly*optics*).

In this article, we outline (meta) theoretical and research-practical ways of engaging with these polycontextural constellations—with the implementation of ESD in primary schools serving as the focal case. Drawing on qualitative data, we reconstruct what appears on the surface as the diffusion of a political program into German primary schools as a chain of intersystemic translation processes. To engage adequately with the phenomenon in its polycontextural complexity, a reflexive analytical perspective is required that enables analysis beyond the constraints of contexture-specific observer positions. This, in turn, necessitates a meta-theoretical engagement with the analytical tools themselves: not merely applying theory to the empirical object, but reflecting on how the choice and use of theoretical concepts shapes what can be seen in the first place. From this perspective, ESD can be treated not monoperspectivally—as either an educational concept or a political agenda—but as a fluid phenomenon that is generalized, specified, transferred, and redefined across different levels and contexts. The *first aim*, accordingly, is to explore and explain polycontextural complications that arise along the intended implementation path—in this case: from a political agenda to its concrete realization in classroom interaction. The *second aim* is to accompany this exploration at a meta-level: to reflect on the iterative interplay between theorizing (Swedberg, 2014) and systems-theoretical empirical analysis—from observing organized interactions to developing a theoretically generalized vocabulary. Taken together, these two aims account for the article’s distinctive twofold analytical design.

We begin with a “prelude” on theoretical observation and develop a *meta-theoretical heuristic* that—in the sense of a “heuristic of the heuristic”—allows us to differentiate and structure processes of theoretical and empirical enrichment in phases, according to varying degrees of generalization. On this foundation, the next section outlines the development of a (case-specific) *systems-theoretical heuristic*, which unfolds in five stages. First, we (re) construct the field of inquiry in polycontextural terms, drawing on basic social-ontological concepts. Second, we situate the phenomenon at the level of theory of society within this reconstructed field, culminating in a general theorization of translation cascades across the identified system and coupling constellations.^1^ Third, drawing on qualitative interviews and ethnographic data from three school development processes, we explore specific interorganizational translation processes between primary schools and consulting organizations along these cascades. Fourth, we distill these findings into two distinct polycontextural complications that provide an explanation for the observed translation blockages. Fifth, we examine the relationship between the two polycontextural complications and their broader generalization for intersystemic transformation processes. In the conclusion, we address how meta-theoretical considerations and systems-theory-informed empirical research relate across the two heuristic levels of the analysis.

## 2 *Prelude*: observing theoretically

When considering how the complex task of implementing ESD can be grasped, it is important to note that even the initial observation—the first delineation of the object—is already liable to be shaped by *praenotiones*, substituting the things themselves with the phantoms of everyday understanding (Durkheim, 1982). Understanding the social world adequately requires resisting the tendency to privilege diffuse, flexible notions of reality over more epistemically controlled forms of articulation. Given that everyday language is “so abundantly rich in possibilities that controlled theoretical reflection becomes impossible” (Anicker, 2026, p. 217), it proves productive to treat theories substitutively as “guiding assumptions for observation” (Kalthoff, 2008, p. 12). The central task is to recognize, reflect upon, and render explicit the theory-laden and theoretically conditioned character of empirical observations (Hirschauer, 2008, p. 167), so that it can subsequently be brought under analytical control. Rather than starting from an everyday notion of “organization” and using it as a template for identifying organizational processes, everyday language is bracketed in favor of a theoretical (re-)definition (Anicker, 2026).^2^ Observation thereby gains in precision while acquiring a reference frame that enables controlled distinctions. Deviations or case-specific variations can then be described relative to this reference, allowing new insights to be systematically situated in relation to what is already known. Accordingly, a dogmatic deployment of a theory constitutes a deliberate theoretical decision in the sense of a *reflexive reductionism*. As will be shown, only then does the strength a theory offers beyond explanation yield transparency regarding the conditions under which it holds and can be generalized. Gaining new theoretical insights is therefore, paradoxically, best achieved “the more dogmatically one proceeds, for dogmas are characterized by a sharp rejection of alternatives and thereby provide a correspondingly precise observation of those alternatives” (Baecker, 2016, p. 7). If theory is assigned such a controlling function, one must nonetheless recognize that sociological theory itself is a product of social practice. Accordingly, the controllability of observation must also be reflected in light of the social situatedness of theory—whether with respect to its case-specific grounding (Hirschauer, 2008) or to the broader habitus of the theorist (Bourdieu, 2020). Since this reflexivity can itself be rendered explicit within theoretical discourse, the heuristic use of sociological theory enables a shift from an intuitive view of the world toward a reflexive, theory-informed mode of observation. Only then does it become possible to break with the “illusion of common sense” (Bourdieu, 2025), to see beyond what one already takes for granted.

Yet how can observation be rendered capable of “surprising in a controlled manner” (John et al., 2010, p. 326) under conditions of strong theoretical embedding, without falling into a logic of subsumption that merely confirms the theoretical concepts guiding observation as hypotheses? If the aim is to develop *a sensitive, visibilizing, theory-laden heuristic* that ensures both openness and control in exploring the field—and that is oriented above all toward society-theoretical considerations—then the preliminary (meta-theoretical) heuristic reflections required for its development call for no less rigor than the (case-specific) heuristic itself. With a view to systematizing the procedure, we distinguish phases of the research process along the gradient of the intended “level of generalization” (Luhmann, 1972, p. 156), differentiating between *negative* changes in the level of generalization (empirical enrichment) and *positive* changes (theoretical enrichment).

*Empirical enrichment* means gathering theory-informed empirical observations. The preliminary theoretical framework thereby serves a dual function as an “orienting device.” First, in the sense of “sensitizing concepts” (Blumer, 1954), with the initial focus on unidirectional use. Social ontology and society-theoretical constellations thus provide a “general sense of reference and guidance in approaching [the] empirical phenomenon […] and merely suggest directions along which to look” (Blumer, 1954: 7). Second, the framework also serves a methodological function: assumptions about social entities already determine which methods are appropriate for capturing communicative processes and the kinds of inferences observation can support (Vogd, 2009). Accordingly, we reconstruct the object in a controlled manner by gradually lowering the level of generalization and increasing the degree of specification, moving toward fine-grained, situationally sensitive analysis.

*Theoretical enrichment* sets in once empirical enrichment reaches a state of saturation and the new insights must be related in theoretical-technical terms. Anicker (2026, p. 233) refers in this regard to a combination of (social-ontological) *building* and (society-theoretical) *placement*. From a differentiation-theoretical perspective, this means placing the observed phenomena within a broader societal configuration and thereby increasing the level of generalization. In the course of theoretical enrichment, the employed society-theoretical sensitizing concepts now function as reference points for further theoretical constructions: observations become “means of revising the concepts” (Blumer, 1954, p. 8).^3^ The result of this theoretical enrichment process is a heuristic instrument that is not introduced into the field as “pre-empirical, i.e., quasi-transcendental” (Nassehi, 2008, p. 96), but rather emerges through empirical and theoretical enrichment, thereby equipping the analysis for the steps that follow. In the remainder of this article, we draw on these conceptual considerations for the development of a concrete systems-theoretical heuristic for analyzing the implementation process of ESD.

## 3 Developing a systems-theoretical heuristic

Given these considerations, a theory is required that can grapple reflexively with polycontextural constellations while guaranteeing sufficient reflexivity at the meta-theoretical level. Luhmann’s systems theory initially appears a suitable candidate—yet accumulated objections raise doubts about its suitability for analyzing dynamic transformation processes. Before turning to the development of the heuristic, we discuss two central critiques that initially seem to challenge the empirical observation of transformation processes (for an overview of the main objections, see Witte, 2025, pp. 148–152). From our perspective, however, these critiques can not only be met but productively reframed as analytical resources for the research design.

The first critique of *conservatism* has been raised since the theory’s earliest reception. Critical theory in particular has accused it of (uncritically) affirming established social formations—a charge linked to its second-order observational stance (Osrecki, 2015, pp. 229–233). Despite the further development of the debate since the 1970s, a persistent line of critique targets structural functionalist conceptions (recently: Mayntz, 2024; Strohmeier, 2025). Unlike Durkheim, however, Luhmann does not understand functional differentiation as an efficient division of labor. Rather, he conceives it as the polycontexturality of operationally closed, self-referential systems (Luhmann, 1995, 2013, pp. 1–9). The social whole can therefore only be conceived as a “society without a top and without a center” (Luhmann, 1990b, p. 16). The critique is reinforced by arguments targeting what one might call the theory’s *Zeitkern* (Benjamin, 2023, p. 578; Mayntz, 2024, p. 142), for example, refers to its historical situatedness, situating it within a specific postwar social formation and questioning its contemporary explanatory power.

For our purposes, this critique can be not only addressed but also turned into analytically productive insights in two respects. First, they concern the society-theoretical perspective that enables second-order observation. While, for example, education, as part of the educational system, produces self-descriptions whose knowledge base is constrained by the “premises of pedagogical self-description” and “normative-pedagogical expectations” (Meseth, 2011, p. 182), a theory of society can provide more distanced forms of (second-order) *other-descriptions* (*Fremdbeschreibung*). Educational scholarship has productively focused on aspects related to schooling and education—for instance, examining the conflicting constellations between reform and transformation approaches and the persistent “grammar of schooling” (Tyack and Tobin, 1994; see also Budde and Blasse, 2023)—yet tends to do so predominantly from a school-theoretical vantage point (see, e.g., Lischka-Schmidt, 2024; Lange et al., 2026). Systems-theoretical theory of society, combined with the theoretical-technical decision in favor of a dogmatic use of theory (reflexive reductionism), promises to open up a novel, reflexive, and complementary perspective on such phenomena. Second, employing a seemingly conservative theory of society for the analysis of dynamic transformation processes creates a productive *contrast* that enables a shift in perspective. Rather than presupposing transformation as a baseline condition—as much of the sociology of transformation and innovation tends to do—we take the fundamental improbability of intersystemic transformation as our analytical point of departure. Accordingly, we do not deploy systems theory subsumptively but a sensitive heuristic that, through recursive engagement with the empirical field, brings theoretical blind spots into view and incorporates them into the analysis.

The second critique concerns the assumption of *operational closure* in autopoietic systems and the related claim that this prevents adequate analysis of the heterogeneous entanglements of societal domains and the hybridity of social constellations (Knorr Cetina, 1992; Münch, 1995). We argue that, from the perspective of functional differentiation, such relations can indeed be analyzed as *polycontextural complications*. Moreover, certain transformation blockages only become visible once operational closure is assumed across divergent horizontal and vertical couplings.

**Figure 1:**
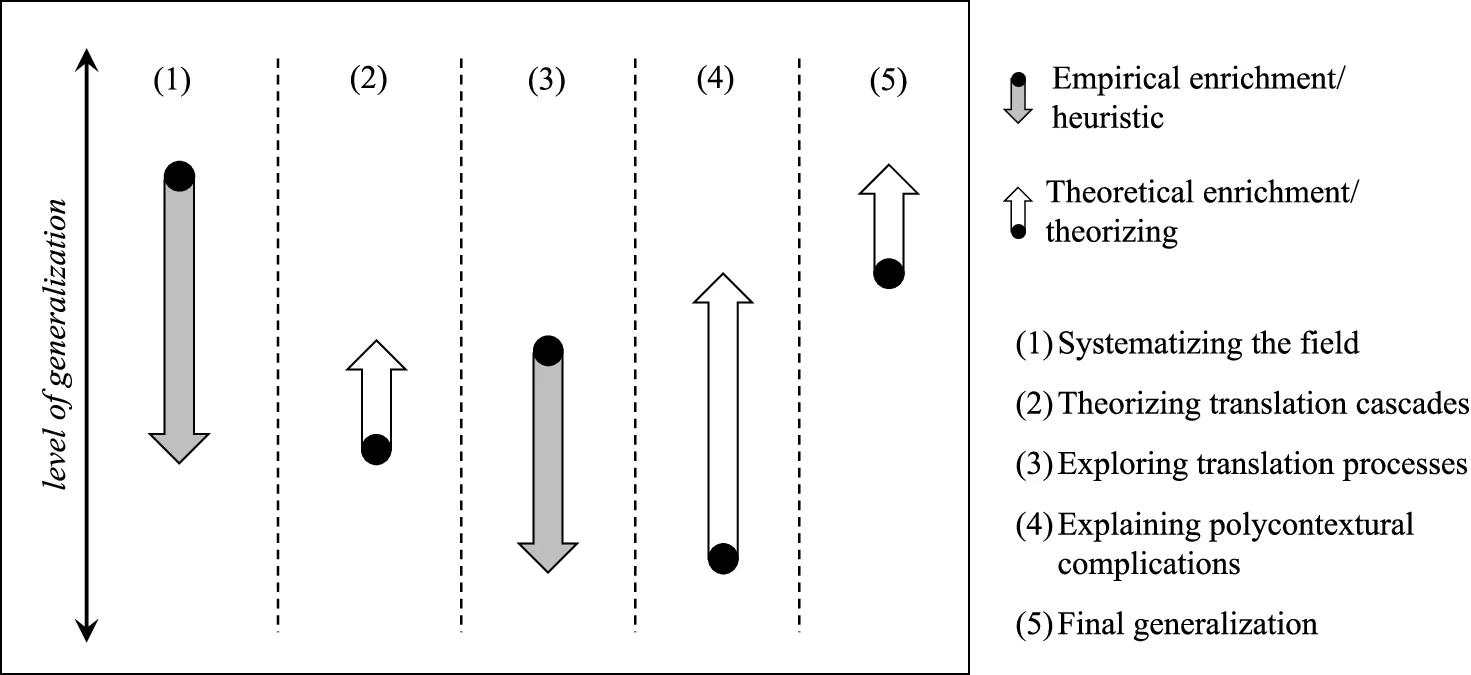
Phases of the iterative-oscillating research process (meta-theoretical heuristic).

Against this background, these critiques prove productive rather than limiting, serving as structuring reference points for the further development of a heuristic to unpack polycontextural complications. Drawing on our meta-theoretical heuristic, with its distinction between *negative* changes (empirical enrichment) and *positive* changes (theoretical enrichment) in the level of generalization, we develop five consecutive phases of an iterative-oscillating research process that together constitute the systems-theoretical heuristic (displayed in Figure 1).^4^ These five phases structure the following analysis.

### 3.1 Systematizing the field

A society-theoretical application of systems theory in empirical research cannot be limited to functional differentiation. As Luhmann puts it, “*before* any consideration of system differentiation, one must ask about different levels of *system formation*” (Luhmann, 2019a, p. 61). To make a phenomenon such as the implementation of ESD in German primary schools amenable to sociological analysis, we therefore begin by systematizing the field—understood as a phase of empirical enrichment that lowers the level of generalization (see Figure 1)—along the levels of society, organization, and interaction (Luhmann, 2005, pp. 9–24; Luhmann, 2021, p. 277; Baecker, 2021; Kron, 2025; Schmalen and Kron, 2026). This vertical axis provides the basis for reconstructing horizontal differentiations between systems.

Assuming polycontexturality as the loss of a unified societal “*EINS*” (oneness) (Fuchs, 1999b) implies that empirical analysis must engage with a multiplicity of heterogeneous “contextures” (Günther, 1979) through which the phenomenon is constituted. The empirical use of theory of society thus requires a basic (re)construction of the phenomenon (in Anicker’s terms: building and placement); what matters here is the point of reference from which explanation proceeds: “a placement-explanation perspective explains something through the *actual* relations it has in a given society” (Anicker, 2026, p. 234). Placement, in this sense, situates the phenomenon within a historically specific societal formation and thereby makes its relations intelligible. As a preliminary step, we begin with the social-ontological building process. In our case, this involves tracing the emergence of ESD in Germany—situated briefly within its international origins—and reconstructing its specific “formation story” (Hirschman and Reed, 2014) in relation to the implementation process under study, especially its education policy dimensions.^5^

#### 3.1.1 Society

From a systems-theoretical perspective, society can be defined as the “totality of all social communications that can be expected” (Luhmann, 1995, p. 392). As outlined above, we approach this level through the lens of differentiation theory, as a coexistence of divergent autopoietic subsystems, which process the world according to their respective binary codes and thereby generate society-wide structures of meaning. Programs complement these codes by specifying the “given conditions for the suitability of the selection of the operations” (Luhmann, 1989, p. 45). It is precisely this distinction between code and program that enables what appears to be a paradoxical combination of operational closure and openness to the environment that characterizes functional systems.

The emergence of education oriented toward sustainability is commonly traced back to the 1960s. In Western academia, studies on the ecological limits of the planet attracted growing attention (Hardin, 1968; Meadows et al., 1972), while political movements and states in the Global South drew attention to the environmental consequences of dominant models of development (Robottom, 1991). From a systems-theoretical perspective, these scientific insights can be understood as environmental irritations to political communication—produced by science operating through the code truth/untruth and generating problem descriptions that become relevant for politics not as truths, but as premises for collectively binding decisions. In this context, UNESCO convened the first intergovernmental conference on the biosphere in Paris (1968). Within a largely natural-science-driven discourse, environmental education (EE) emerged as a way to integrate environmental issues into school curricula (Pavlova, 2013). The Brundtland Report (1987) and the UN Conference on Environment and Development in Rio (1992) marked a shift in the UN’s approach by linking environmental and development concerns, thereby reframing EE as Education for Sustainable Development (ESD), increasingly oriented toward human development (Pavlova, 2013).

These international developments found resonance in Germany as well. Following the Rio Conference and Agenda 21, the Federal Government-Länder Cooperation (BLK) launched the program “21 – Education for Sustainable Development” in 1999, with the aim of integrating it into regular school practice. The 2002 Johannesburg World Summit further reinforced this trajectory, calling for the integration of sustainable development into education systems “in order to promote education as a key agent for change” (United Nations, 2002) and launching the UN Decade of ESD (2005–2014). In 2004, these developments culminated in the German National Action Plan, unanimously adopted by the Bundestag (German Bundestag, 2004b, p. 10757), which gave ESD the status of a binding policy program. Politically, the “promotion of basic education [was positioned] as a central dimension of a [broader] sustainability strategy” (German Bundestag, 2004a). With the Agenda 2030 and the Sustainable Development Goals (SDGs), ESD was further reframed as a key organizing framework. In Germany, a renewed National Action Plan was adopted in 2017 with 130 targets and 349 recommendations aimed at structurally anchoring ESD across the education system (Federal Ministry of Education and Research (BMBF), 2017). At the international level, the UNESCO “ESD 2030” program (UNESCO, 2021) continues this trajectory, outlining how global challenges are to be addressed through education. In this sense, ESD appears as a political program that, among other things, processes scientific irritations, translates them into binding decision premises through its binary code, and directs them toward the education system in the form of structuring expectations.

#### 3.1.2 Organization

Organizations constitute themselves as social systems through recursively closed decision communication (Luhmann, 1972, 2002, p. 159; Besio and Tacke, 2024). Decisions serve the absorption of uncertainty: “in a world that is opaque to them, they transform uncertainty into certainty” (Luhmann, 2018, p. 168). In doing so, they transform a closed past in which no changes are possible into an open alternative awaiting decision, and at the moment of decision, replace what remains contingently open with determinate outcomes. At the same time, they define a distinction that serves as an orientation point for observing the future and for bringing it into the decision process in a structured manner. Organizations rely on decision programs as selection aids. These programs either orient decisions toward organizational goals (*purposive* programs) or establish conditional if-then relations for processing specific inputs (*conditional* programs) (Luhmann, 2018, p. 213). Within the implementation of ESD, this framework enables the identification of different organizational systems and the reconstruction of system-specific chains of decisions. A key aspect of this exploratory enrichment is the reflexive and controlled use of preliminary theoretical concepts. Pre-theoretical distinctions, such as the organizational boundaries of schools, may serve as initial heuristic guides in a Durkheim (1982) sense discussed above, but are treated here as organizational self-descriptions. As organizations adapt to their environment, ESD is processed across organizational systems through chains of decision operations.

In the organization of the primary school, implementation initially takes the form of the formal decision—typically taken by school leadership or a steering group—to re-specify political irritations and incorporate ESD as a development priority in the school program. This triggers further decisions regarding curriculum adaptation, new formats, staff training, time allocation, and working groups. Compared to other organizations in the educational field, primary schools are characterized by specific conditions, i.e., decision premises—for instance, a typically small number of students and staff, as well as a particular resource endowment (Timm and Barth, 2021; Lange et al., 2026). Each decision reduces uncertainty by excluding alternatives while generating new decision premises, for instance regarding responsibilities, evaluation, or documentation. The same applies to the decision-making processes of organizations that, by virtue of their purposive programs, can be described primarily as “political organizations.” In the context of ESD implementation, they can be reconstructed as recursively linked decision structures organized according to center//periphery differentiation (Luhmann, 2000, p. 244) and specific decision premises. At the political center—such as federal or state ministries—decisions typically take the form of vague, consensus-oriented programs that function as generalized decision premises for subordinate organizations. These are specified in strategies, funding lines, or curricular frameworks, creating selective openings for subsequent decisions, for example, in the form of application rules, resource allocation, and (vague) thematic priorities.

The transmission and transformation of these programs proceed through formally structured communication channels, particularly along administrative hierarchies such as regional education authorities. These function as autonomous organizations that do not merely implement programs but actively specify them through their own decisions. Communication channels define the conditions under which decisions can connect to further decisions: decrees, guidelines, and advisory communications stipulate the conditions under which schools may apply for funding and which forms of implementation are considered appropriate. This produces further decision chains, for instance, when the interpretation of funding criteria by supervisory education authorities triggers additional selection decisions at the school level. At the “political periphery”—in particular school conferences—these pre-structured decision premises are taken up under conditions of heightened irritability to environmental expectations and translated into organizational decision-making. As a formal body integrating different perspectives (school leadership, teachers, parents, students) through membership, school conferences transform political-administrative programs into collectively binding decisions. For instance, funding opportunities may lead to decisions on concrete investments (e.g., sustainability-related infrastructure or projects), which in turn generate further decisions on resource allocation, use, and curricular integration.

This produces a recursive structure of decisions that makes ESD legible as an organization-internal construct. Decision programs function as selection aids: purposive programs may orient measures toward the objective of “sustainable school development,” while conditional programs specify the conditions under which certain steps are to be taken and the means to be employed. At the organizational level, ESD thus appears as a sequentially stabilized decision structure.

#### 3.1.3 Interaction

Interactions complete the vertical axis of system formation; they constitute social systems whose selectivity and boundary formation rest on mutual perception among co-present participants (Luhmann, 1995, pp. 414–415). In the context of ESD, classroom interaction forms the focal point of implementation, as the “core of the differentiated education system” (Luhmann, 1986, p. 97). It is here, ultimately, that the politically mandated “transmission of basic skills and factual knowledge about the interrelations between humans, nature, and technology” (German Bundestag, 2004a) is expected to materialize. Transformation efforts, therefore, are directed at different structural dimensions of the classroom interaction:

In the *social* dimension of meaning, this concerns the education-specific asymmetric teacher-student role structure, which shapes classroom interaction strongly while remaining situationally interpreted and continuously (re)enacted (Luhmann, 2002, p. 108; Drepper and Tacke, 2024, p. 415). ESD-focused reform initiatives aim to reconfigure this relation, in particular by decentering the teacher role, as expressed in notions such as “learning companion” or “coach” (Rasfeld, 2021, p. 34). In the *substantive* dimension, transformation efforts focus on sustainability-related topics that are increasingly integrated into classroom practice. At the same time, emphasis shifts from traditional subject content toward future-oriented, transversal competencies cutting across established curricular and disciplinary structures (Fullan and Langworthy, 2013). The comparatively weak subject differentiation characteristics of primary schools in contrast to secondary education provides a structurally more favorable basis for such cross-curricular endeavors (Lange et al., 2026, p. 53). In the *temporal dimension* of the classroom interaction, ESD formats operate both at the level of organizational “periods”—for instance, the “FREI-Day” substitutes project-based phases for parts of regular instruction (Rasfeld, 2021)^6^—and at the level of interactional “episodes” (Luhmann, 2002, p. 108): The redefinition of the teacher as a learning companion implies that students increasingly decide when and at what pace to engage with specific topics. Finally, the *spatial dimension* concerns the organization of classroom interaction in physical terms. While Luhmann emphasizes spatial closure as a condition for autonomy and control over topic selection (Luhmann, 2002, p. 107), ESD approaches promote more flexible learning environments, including variable seating arrangements and decentralized learning spaces that extend beyond the classroom (Martínez-Ramos et al., 2021; Peng et al., 2022; for design perspective see also Bosch, 2018). In organized classroom interaction, the decision structure of ESD thus manifests—re-specified—as a social, substantive, temporal, and spatial constellation of meaning at the level of interaction.

### 3.2 Theorizing translation cascades

The preceding modelling of the field—essentially an identification of relevant social systems across different levels of complexity, informed by social ontology—is a necessary but not sufficient step in a society-theoretical account of an empirical phenomenon. If ESD is to be analyzed not only “from within” but also in terms of its relational entanglements “from without” (Anicker, 2026), a second step is required: the (re)constructed elements must be related to one another through a process of (society)theoretical enrichment that raises the level of generalization (see Figure 1). In line with the two axes of system levels and system differentiation, the relations to be examined in the implementation of ESD as “ecological communication”^7^ are here conceptualized as vertical and horizontal couplings. The *vertical* axis structures the analysis sequentially: first, we situate the implementation process in the relation between society and organization; second, we extend this relation to the level of interaction. *Horizontal* couplings, by contrast, cut across these steps and are considered throughout.

Organizations and society are constitutively related. The continued reproduction of modern society’s forms of autopoiesis depends on the operations of organizations. At the same time, organizations draw on the structures of functional systems in making decisions: binary codes such as payment//non-payment, legal//illegal, or able//unable, along with symbolic media such as money, all serve to shape organizational decision-making. In this sense, organizations continuously reproduce societal structures internally while also relying on the structures available in their environment: “The distinction between system and environment, which arises with the formation of organization, engraves itself, so to speak, into society. There is society on both sides of the system boundary” (Luhmann, 2018, p. 318). Yet the expectations conveyed by functional systems remain too abstract to be directly incorporated into organizational decision-making. Rather, they require a “*respecification* of those indeterminacies that are unavoidable at the level of societal functional systems” (Luhmann, 2002, pp. 164–165).

While Luhmann assumes that organizations can in most cases be assigned to a single functional system (Luhmann, 2000, pp. 228–274; Luhmann, 2018, p. 333; Luhmann, 2019b, p. 230),^8^ subsequent systems-theoretical approaches highlight forms of “multireferentiality” (Wehrsing and Tacke, 1992; Lieckweg and Wehrsig, 2001), “multifunctionality” (Will et al., 2018), or “polyphony” (Andersen, 2003) of organizations. In order to theorize how organizations incorporate divergent societal logics into their decisions, Besio and Meyer (2020) introduced the concept of “re-combination”. We focus on a specific form of re-combination conceptualized as “translation,” through which organizations “recombine and reconcile different systems’ requests” (Besio and Tacke, 2024, p. 1367). This situates the analysis within a strand of differentiation theory that foregrounds “practices of translation [as] the basic mode of operation of modern societies” (Nassehi et al., 2019, p. 197). Here, the focus shifts from problems of societal integration to “translation conflicts” emerging in the course of implementing societal solutions (Mölders, 2025, pp. 198–203; see also Renn, 2025). Organizations appear here as sites where such conflicts are negotiated and addressed. Building on Nassehi’s characterization of the German Ethics Council as a “translation agency” (Nassehi, 2021; see also Nassehi et al., 2019), Mölders (2025, p. 209) proposes extending this concept from the level of organized interaction to organizations more generally (see also Lutz and Mölders, 2025).

Schools face this dual requirement of re-specification and translation in implementing ESD. The National Action Plan offers a telling illustration: one of its 130 goals calls for embedding ESD’s pedagogical principles in teaching and school development [Federal Ministry of Education and Research (BMBF), 2017, p. 32]. To this end, it proposes developing teaching practices and integrating ESD into school-based curricula, including model curricula, while leaving implementation deliberately open: “Schools decide, under their own responsibility and within their remit, how to implement ESD in in-school and out-of-school learning as well as in school life and everyday school practice” [Federal Ministry of Education and Research (BMBF), 2017, p. 32]. This produces a *double vagueness* at the organizational level. Horizontally, ESD—anchored in the SDGs, specified as a “framework for addressing immense challenges” [Federal Ministry of Education and Research (BMBF), 2017, p. 7]—appears as a heterogeneous bundle of goals, drawn from divergent but equally weighted societal expectations, requiring schools to re-combine and translate them. Vertically, vagueness emerges in the relation between functional systems and organizations: translation becomes operational only to the extent that abstract expectations are re-specified in organization-specific decisions.

Because such processes demand considerable time from individual schools, they are typically relegated to the domain of school development, beyond the school’s core tasks; at the organizational level, structures such as inset days^9^ are established to accommodate these processes. In this context, consulting organizations enter the scene as “translation agencies” (Mölders, 2025). They promise to make manageable the crisis constellations omnipresent in functionally differentiated societies characterized by polycontextural complexity (Fuchs, 2004, p. 245), and support schools in navigating the challenges that arise in translating (educational) policy programs. By preparing ESD programs for implementation and providing process support, they seek to facilitate a further re-combination, which is precisely the purpose for which they are introduced. Accordingly, in what follows we distinguish between *first-order* translation (intraorganizationally performed by schools) and *second-order* translation (interorganizationally performed by schools and consulting organizations).

Interaction, as the third type of social system, stands in a reciprocal relationship with both society and organizations. Interaction is also central to the implementation of ESD: “All social action must, factually, pass through this needle’s eye and is deformed by the inherent laws of interaction systems” (Luhmann, 2019a, p. 67). Within interaction, functional systems provide orientation through abstract definitions of the situation (Kron and Laut-Berger, 2022, pp. 142–146). As distinct contextures, they constitute structured horizons of expectation that define “fields of probability” for possible connections (Vogd, 2009, p. 113). Given their limited capacity to generate structure, interactions necessarily rely on such societal frameworks, enhancing their capacity for specification and differentiation (Luhmann, 2019a, pp. 68–69). Functional systems also shape interaction at the level of programs. Curricula, for example, specify which topics are to be addressed within given time frames—typically mediated through organizational re-specification and translation. Organizations, in turn, shape the conditions of interaction; for example, schools ensure through organizational decisions that classroom interaction begins and ends at fixed times (Luhmann, 2002, p. 164).

Neither functional systems nor organizations are able to determine interaction. Rather, interaction is an emergent social system that reproduces itself according to its own complexity—as a “unity of routine and contingency, of order and disorder”—and thus operates under conditions of fundamental uncertainty, oriented toward an open future (Luhmann, 2002: 104, 109). In recombining societal expectations and functions, organizations must “respecify them to such an extent that behavior in immediate [classroom] interaction can connect to them” (Kieserling, 1999, p. 340). Although classroom interaction takes place as organized interaction within the school, the organization itself cannot program or control its internal dynamics; interaction between teachers and students does not operate in the communicative mode of decision (Luhmann, 2002, pp. 161, 164). This highlights the limits of both societal and organizational intervention in implementation processes such as ESD: classroom interaction ultimately “follows its own course and the history it produces” (Luhmann, 2002: 164).

**Figure 2:**
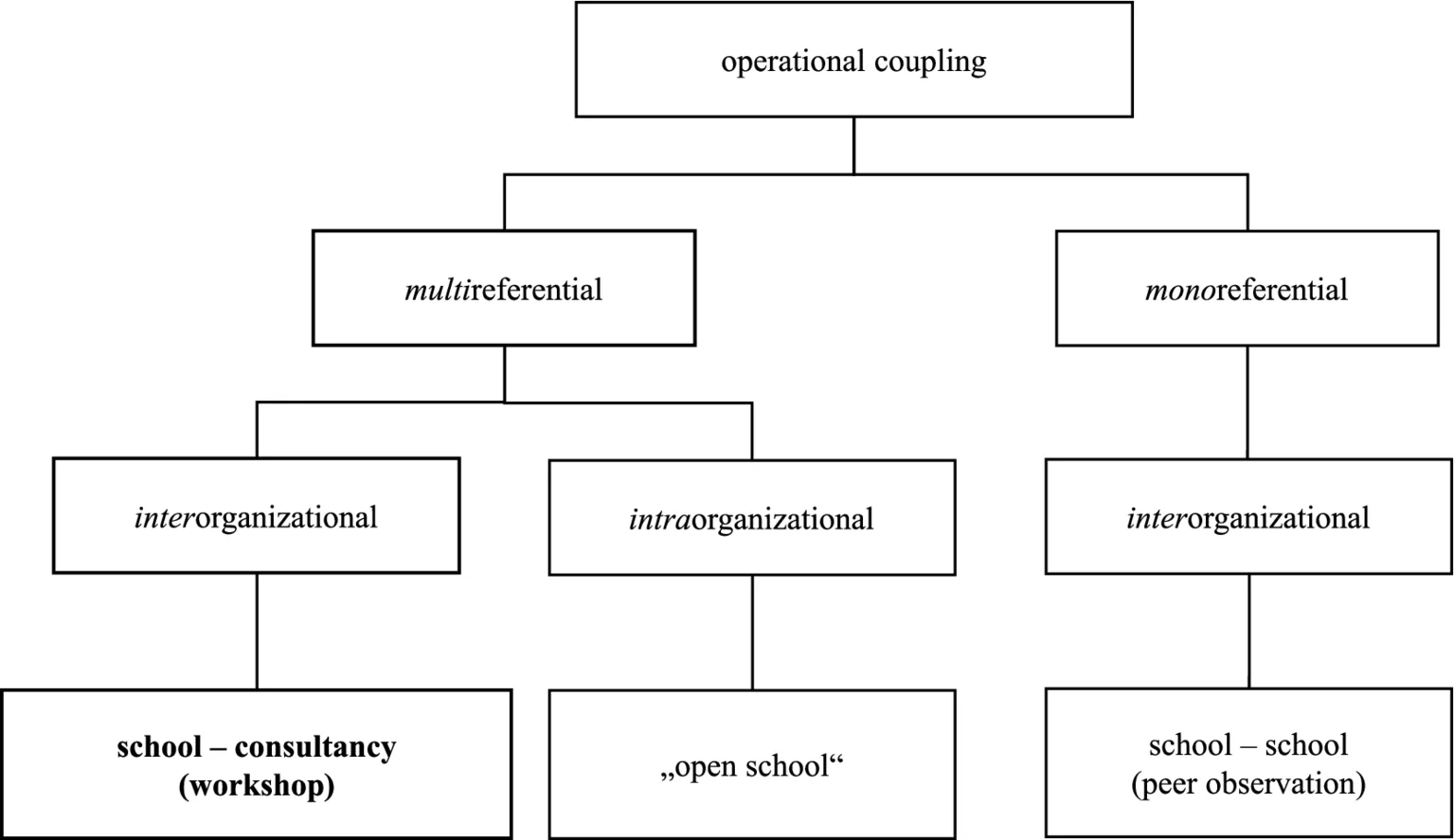
Translation cascades.

Rather than confining the empirical analysis to organizational self-descriptions at face value—for example, the sustainability-oriented profile of “School in Transition” (“Schule im Aufbruch”)^10^ or concepts such as the “whole school approach”^11^—we now turn to the *sites of translation*. Re-combination will not be taken as an already operative fact; rather, the subsequent analysis empirically traces concrete implementation events and reconstructs the corresponding translation cascades in terms of the systematization developed above. Against this backdrop, Figure 2 presents the analytical structure that guides the theoretically enriched placement of translation formats. Each translation format represents an “operational coupling” (Luhmann, 2004, pp. 381–382)—a momentary linkage between system and environment at the level of a single event, in which the same occurrence is processed through each system’s own recursive logic, thereby generating divergent, system-specific meanings. One such format is the workshop, in which school members and “translation professionals” from an ESD-specialized consultancy participate together. As a translation format, the workshop typically forms part of a multi-stage school development process and, in line with the analytical structure presented above, represents an multireferential and *inter*organizational translation process.^12^ Its purpose is to translate external expectations—and logics initially external to the education system—into organizational programs through facilitated re-specification.

### 3.3 Exploring translation processes

With the society-theoretical modelling of the field and the subsequent placement of the phenomenon within the structure of relations along vertical and horizontal system(—level) differentiation, ESD is sufficiently delineated for the empirical analysis of translation processes. We now focus on one node in the cascades where re-combination is centrally negotiated: the multireferential, interorganizational development process involving a primary school and a consulting organization (see Figure 2). According to the theoretically enriched reconstruction, this coupling process translates ESD, as the outcome of political decisions and a complex of societal expectations (horizontal coupling), and re-specifies it (vertical coupling).

To better understand this process, the theoretical reconstruction is now to be empirically enriched through a focused examination of the “needle’s eye” of concrete interactions—the level of generalization decreases considerably [step (3) in Figure 1]. For this purpose, participant observations and ethnographic conversations are drawn upon, collected during repeated visits to three German primary schools during lessons and at an inset day (data collection period: October to December 2024). In addition, qualitative interviews were conducted with teachers, school leaders, after-school staff, and consultants (*n* = 7, data collection period: November 2024 and February 2025). As the interviews were conducted following the participant observations, it was possible to address and reflect on specific situations and impressions from the workshop, supplemented by peer observations in the case of the two principals. This approach is based on the idea of theoretical and selective sampling (Glaser et al., 2010; Misoch, 2019, pp. 204–212): the saturation of empirical enrichment is thereby determined by the preceding theorization. In cooperation with a consulting organization, a transdisciplinary seminar was furthermore organized, in which consultants, school leaders, teachers, and actors from the district authority participated (*n* = 13, June 2025). During the seminar, initial findings were presented and discussed in focus groups.

A concrete, empirically observed school development process serves in what follows as the primary case study. The field notes and transcripts were analyzed using qualitative methods of frame and sequence analysis (Vogd, 2005, 2009; see also Goffman, 1974). The (reconstructive) analysis is thus not interested in “subjectively intended meaning as the basis of social action, but exclusively in the recursive interconnection of self-referentially related communicative events,” and consequently focuses on “communicative sequences according to their internal regulatory routines and processing structures and structured processes” (Nassehi, 1997, pp. 138, 149).

For the operationalization, we draw on the metatheoretical considerations from section 2: In line with a society-theoretical perspective, the analysis requires continuous relating of abstract macro-structures and situational micro-elements. The analysis therefore focuses on the use, updating, and (re-)specification of these “macro-structures,” observable in interaction as “*Translate*”—products of translation processes—that manifest as situational arrangements of heterogeneous contextures (Renn, 2018, pp. 169–170, 210). Such specification processes are operationalized in the course of observation through the *empirical enrichment* of *open* concepts toward *definitive* concepts (Blumer, 1954; Kelle and Kluge, 2010, p. 30). In our case, this concretely means the systematization of the field and its theorization (Figure 1) function as sensitizing lenses guiding empirical observation. Without undermining the principle of openness upon entering the field, we explicate and reflect on the vague, unspecified lenses during observation, in line with the above heuristic. In preparation for the data collection, field notes and interview guides were prepared incorporating *sensitizing* concepts derived from *theoretical enrichment* (see section 2).^13^

In the following sections we focus on an exploration of first- and second-order translation processes. This raises, on the one hand, questions concerning the relation between the ESD program to be translated and the established educational program of the school, as well as polycontextural complications in its implementation that require consultant-supported change processes (*first-order* translation). On the other hand, the translation process as constituted through the multireferential, interorganizational development process between the primary school and the consulting organization at the workshop is analyzed (*second-order* translation).

According to the German Federal Ministry for Education, Family Affairs, Senior Citizens, Women and Youth (BMBFSFJ), ESD refers to education that “enables people to think and act in a future-oriented manner”; sustainable education thus goes beyond factual knowledge and fosters “anticipatory thinking, interdisciplinary knowledge, autonomous action, and participation in societal decision-making”^14^. Linked to the SDGs, these vague expectations are initially re-specified in educational research as competencies—subject-specific, methodological, social, and personal—termed “future skills” (Fullan and Langworthy, 2013; Rasfeld, 2021). Whether these future skills can be taught and learned became central to implementation. They are to be acquired mainly through student-driven, self-directed learning and differ markedly from established “classical learning goals,”^15^ through which schooling specifies educational aims as clearly defined content in formal programs (curricula). This form functions as a decision premise for further decisions on teaching formats, timetables, assessments, staffing, and content. The novel expectations of ESD are therefore not defined solely by ecological content. Rather, it aims to enable students to develop reflexive and autonomous perspectives on problems. Its thematic focus includes both explicit sustainability topics and issues beyond ecological, social, or economic sustainability. Central to first-order translation and re-specification is therefore the relation *between* and integrability *of* future skills and classical learning goals. This is explicitly problematized during the pedagogical workshop and then seemingly resolved through various translation practices guided by consultants. Subsequent findings, however, reveal significant blockages in translation and re-specification.

For the workshop, the school leadership invited a consulting organization specializing in school development to support the implementation of ESD. The school is a “classical primary school”^16^ with an open all-day program and a low degree of ESD implementation, representative of most German primary schools.^17^ The ethnographically observed workshop marked the first engagement with ESD. A transformation impulse is nonetheless evident, as demonstrated by the choice of ESD as the theme for the inset day. To date, sustainability has been addressed only in extracurricular project weeks, one-off activities, or after-school clubs within the all-day program (depending on programs designed by after-school staff). Regular school time is strictly standardized spatially, temporally, socially, and curricularly. Guided by prescribed curricula, classical learning goals are taught in whole-class, teacher-led instruction in assigned classrooms. Ecological topics are primarily treated as part of the curriculum in science class, with both subjects and topics predetermined for students.

As outlined above, the workshop as an interorganizational, multireferential development process primarily targets translation and re-specification. The empirical analysis of the external (political) program indicates a *de facto* exclusively *asymmetrical* translation; the aim is to establish operational compatibility with the school’s decision chains and classroom interaction, both oriented primarily to the functional logic of the education system.^18^ In this context, the consulting organization acts as a “translation agency” (Mölders, 2025). The interaction system includes the entire teaching staff (TEACH), after-school staff, and school leadership, while the consulting organization is represented by two members (CON1; CON2). The workshop takes place on school premises and is largely interactive in structure, combining inputs by CON1 and CON2 with group work, presentations, and plenary discussions.

At an advanced stage of the workshop, the central relation between the programs to be integrated in first-order translation is repeatedly problematized (fieldnotes)^19^:

CON1 takes the floor and responds to TEACH3, again addressing the relation between “learning goals” and “future skills”: future skills, CON1 argues, lie “behind” them. The point is thus less to *add* future skills to subjects such as German and mathematics than to “*reflect the underlying* competencies.” […] It is also important to “enable” students. CON2 agrees and adds that students can be “idea generators.” Prompted by these remarks, a teacher adds: “Students can participate and decide.”

The intersystemicity of the programs is figured as a duality by the “translation agents.” This duality implies integrability (or re-combinability) that merely needs to be “reflected,” that is, recognized. The SDGs are then invoked as the reference framework for translation and re-specification. After a 30-min input outlining future skills, the UNESCO, ESD, and the SDGs, participants are asked to name a SDG they consider particularly relevant and compatible with their teaching. The plenary response shows that this transfer proves difficult for teachers; responses are almost entirely absent. Guided by the workshop facilitators, an exercise follows that can be interpreted as a translation practice, essentially based on the controlled alignment of educationally distant and proximate purposes (programs) (fieldnotes):

Colored moderation cards are arranged in a circle on the floor at the center of the seating circle, forming the so-called “education flower,” a pedagogical concept representing different educational domains. CON1 asks participants to position themselves by the domain they best match in their role as teacher or after-school staff, as well as privately. The allocation is quick. In a subsequent reflection, some explain their choices. While some select domains aligned with their subjects—e.g., TEACH6, a mathematics teacher, stands at “mathematical education”—others refer to past, partly unsuccessful educational trajectories or personal interests. TEACH10, positioned at “science and technology education,” reports having initially worked as an engineer. TEACH9 joins many others at “arts education,” noting a strong personal interest in art and past participation in workshops at a museum. […].

CON1 then assigns the 17 SDGs to the ten domains of the education flower. Whereas the earlier question, “Which of the 17 goals are you passionate about?” elicited little response—only two participants answered (poverty: “because I engaged with it during my studies”; life below water: “because children relate to it most”)—each participant is now assigned a specific goal based on their prior self-positioning within an educational domain.

As the observation reveals, participants find it significantly easier to relate their individual understanding of education to a pedagogical than to a political program. The translation agents exploit this functional compatibility by translating the SDGs into a program compatible with teachers et al.

While the workshop interaction system suggests successful re-combination and re-specification through presumed integrability, a contrasting picture emerges beyond the workshop. This appears in classroom interaction where the new programming encounters the established order of organized interaction, in which career value must be generated (in this concrete case, through grading by the end of fourth grade)—as illustrated by an account from TEACH1:

And then you reach a point where *two systems somehow confront each other* and sort of clash. […] You can no longer take certain interests into account; you cannot really do anything with regard to the FREI-Day—*how are you supposed to assess that?* […] You cannot make use of these future skills, you cannot use anything related to sustainability that particular projects have produced. What they have done, *you cannot capture it*. It’s simply not possible, and that’s really unfortunate. That’s where the freedom just ends.

Project-based work (here the “FREI-Day”), intended to foster future skills, encounters systemic limits and escapes established programming—“you cannot capture it.” Rather than the duality figured during the workshop, the relation is presented here as a *dualism*. The teacher speaks of two systems that “somehow confront each other.” Similarly, a school leader and teacher (CON1) describes problems in classroom implementation during an interview:

It’s really difficult to assess whether children develop future skills or not. (…) If I take “critical thinking,” for example, and consider a group of students I teach, I usually know quite well who questions things—who can think critically and who simply accepts things as they are? (.) That still works fairly well. If you ask, “What can you do to save water?”, they have to think critically about it. *Some can do this better than others*. That still works.

Here, too, re-combination—that is, translation and re-specification—appears to encounter limits. It is “really difficult” to *assess* future skills. Notably, CON1, here in her role as a teacher, also participated in the workshop described above, in her role as a consultant.^20^ Even she reverts here, unquestioningly, to the established system logic. The problem is explicitly framed as the measurability of goals. As a solution to resolving this “framing conflict” (Vogd, 2005) between the political program and pedagogical communication, an asymmetrical translation into the established logic of the latter takes place. Measurability—as the basis for fitting into the binary schema of better//worse (“*Some can do this better than others*”)—is presupposed as an unquestioned premise.

Beyond these blockages of first-order translation, the marked discrepancy between the figuration of the translation instances—integrable duality//non-integrable dualism—during and beyond the workshop points to a more general problem of second-order translation that is acknowledged by the consulting organization. In the transdisciplinary seminar, a consultant reports on an experience from a consulting process:

We had two sessions fairly close together, and they told me they had found the last supervision really great. We had talked quite intensively about their organization, and they had tried to discuss this with their supervisors in their own organization. (.) Then some time passed before they could arrange meetings, and although they had found it really great, when they spoke with their supervisors, (.) *they no longer really knew what it had actually been about. They still knew in a general sense, they could talk about it, but somehow it had just dissipated*.

Analogous to the situation described above, the consultant (here assuming an observer position) describes the unavailability of the seemingly successful intervention. The “really great” events during the consulting session “dissipated” beyond the session.

### 3.4 Explaining polycontextural complications

We now turn to explaining the blockages in the implementation process identified in the empirical exploration. In line with our meta-theoretical heuristic, this involves theoretical enrichment and an increase in the level of generalization (step (4) in Figure 1). The implementation of ESD in primary schools was modeled above as a form of organizational re-combination, comprising asymmetrical translation and re-specification of external expectations and vague programs in order to render ESD operational within the school’s decision processes. Given this asymmetrical translation constellation, this presupposes the compatibility of external logics with established programming that structures classroom interaction as the “core of the differentiated education system” (Luhmann, 1986, p. 97). Given the distinction between first- and second-order re-combination, the identified polycontextural complications may be systematized as follows: The dualistic relation between future skills and classical learning goals emerges as a fundamental problem of asymmetrical translation and re-specification (first order) in the organizational implementation of external expectations and vague programs in primary schools, thereby constituting a *first-order polycontextural complication*. To enable first-order re-combination, a consulting organization was involved through a workshop, thus constituting a further process of translation and re-specification (second order). The resulting unavailability of the seemingly successful intervention and re-combination points to a distinct *second-order polycontextural complication*.

#### 3.4.1 First-order polycontextural complications: contingency incongruence

The first-order re-combination constitutes, *de facto*, an asymmetrical translation and re-specification.^21^ The external sustainability logic, concretized in the concept of future skills, is to be integrated *into* the established logic in order to re-combine divergent expectations within the organization. The relation between future skills and classical learning goals thus represents a cardinal point of first-order recombination. The empirical enrichment reveals a dualism structured by the distinction between trivial and non-trivial machines. In the acquisition of classical learning goals, students are treated as trivial machines^22^: given an input—such as a question or task—they are expected to produce a single correct output. By contrast, the concept of future skills presupposes students as non-trivial machines: a (“sustainable”) response to a problem often cannot be derived through the mere application of a memorized rule; the relation between input and output is not bijective; rather, the output comprises an open-ended set of possible solutions. The processing of input thus depends on the psychic system’s *eigenstate*. Whereas this influence of the eigenstate is undesirable within the classical learning model—where individual interests, creative alternatives, and emotional states impede the precise application of memorized rules (Luhmann, 1987c, p. 193)—it is explicitly required and fostered within the framework of future skills.

Insofar as the logic to be translated (future skills) and the existing instructional program (classical learning goals) stand in a *dualistic relation*, the code of the education system appears to dominate classroom communication. The code, according to Luhmann, results from the need to “develop a career, i.e., to construct a sequence of one’s own selection and the selection of others, and signifies the condition of possibility and structural restriction for events connected with this” (Luhmann, 1989, p. 101). Even at the primary level, an evaluative mechanism is required that, at the latest in organizational decisions about transition to secondary schooling, assigns a career value to the fulfillment or non-fulfillment of prescribed learning goals (decision premise). Although the education system, especially in the context of the educational transformation considered here, does not primarily serve to select communication but rather has the function “to change humanity” (Luhmann, 1989, p. 101), a structural differentiation between coding and programming is also present here. Sustainable education is limited by the system’s coding of *able//unable* (or the subcoding *better//worse*).^23^ Transferential couplings and interpenetrations (Münch, 1991), mediated through programs (here curricula), initially enable connections to societal issues: global warming, CO₂ emissions, ocean pollution, and possible solutions are part of the prescribed curriculum, for instance in biology or general studies. This connection, however, corresponds only to the above-described concept of classical learning goals. In order to generate career value, communication produces an artificial, formally binary-coded contingency that is imposed on all programs (Luhmann, 1987b): students *can*, for example, list different greenhouse gases, or they *cannot*. Contingency is thus constituted along the binary code, while excluding further contingency of knowledge itself that cannot be assigned a value. To extend this schematic example: novel alternative approaches to greenhouse gas emissions are systemically excluded from communication or can be addressed only in a restricted manner. Such a “dominance of coding” (Luhmann, 1989, p. 103) accordingly privileges learning models that treat students more as trivial machines. Orientation toward classical learning goals thus secures the integrability of coding and programs. By contrast, learning concepts of sustainable education oriented toward students’ competencies and interests cannot be mapped; as TEACH1 puts it, “you cannot capture it,” since the contingency underlying them exceeds the systemically provided, binary structure of contingency delivered by the code. The first-order polycontextural complication thus resides in an *incongruence of the programmatic quality of contingency*, which hampers asymmetrical translation.

#### 3.4.2 Second-order polycontextural complications: a-addressability and emergence of the intervention system

The empirical findings point to (at least) one further blockage not captured by the explanation of the first order. The intervention, or re-combination, successfully enacted in the multireferential, *inter*organizational workshop proves unavailable beyond the intervention system—the communication remains inaccessible. The duality figured in the workshop and the resulting re-specified translation appear to fail in their transfer to the school and classroom. To understand this, we examine more closely the constitution of the intervention system within which programmatic transformation unfolds. Interorganizational networks presuppose external communicative capacity. Qua decisions, organizations create speaker roles that enable them—uniquely among social systems—to communicate across system boundaries with their environment on their own behalf. Crucial here is the dual relation between organization and society outlined above (Luhmann, 2018, p. 318):

There is society on both sides of the system boundary. The system boundary of the organization can therefore, unlike the external boundary of the societal system, be crossed through communication, even if the organizational system itself is operationally closed on the basis of its own decisions. An organization, thus, always finds society in a double sense: within itself and in its environment.

In line with the social-ontological premises of autopoiesis and observation, it bears noting that external communication, as self-description, remains an internal operation. At the operational level, boundary-crossing communication in the sense of coordination or consensus is excluded; contact with the environment is not possible. Unlike society and functional systems, however, organized systems—as well as psychic systems (consciousness)—are addressable (we return to this).

**Figure 3:**
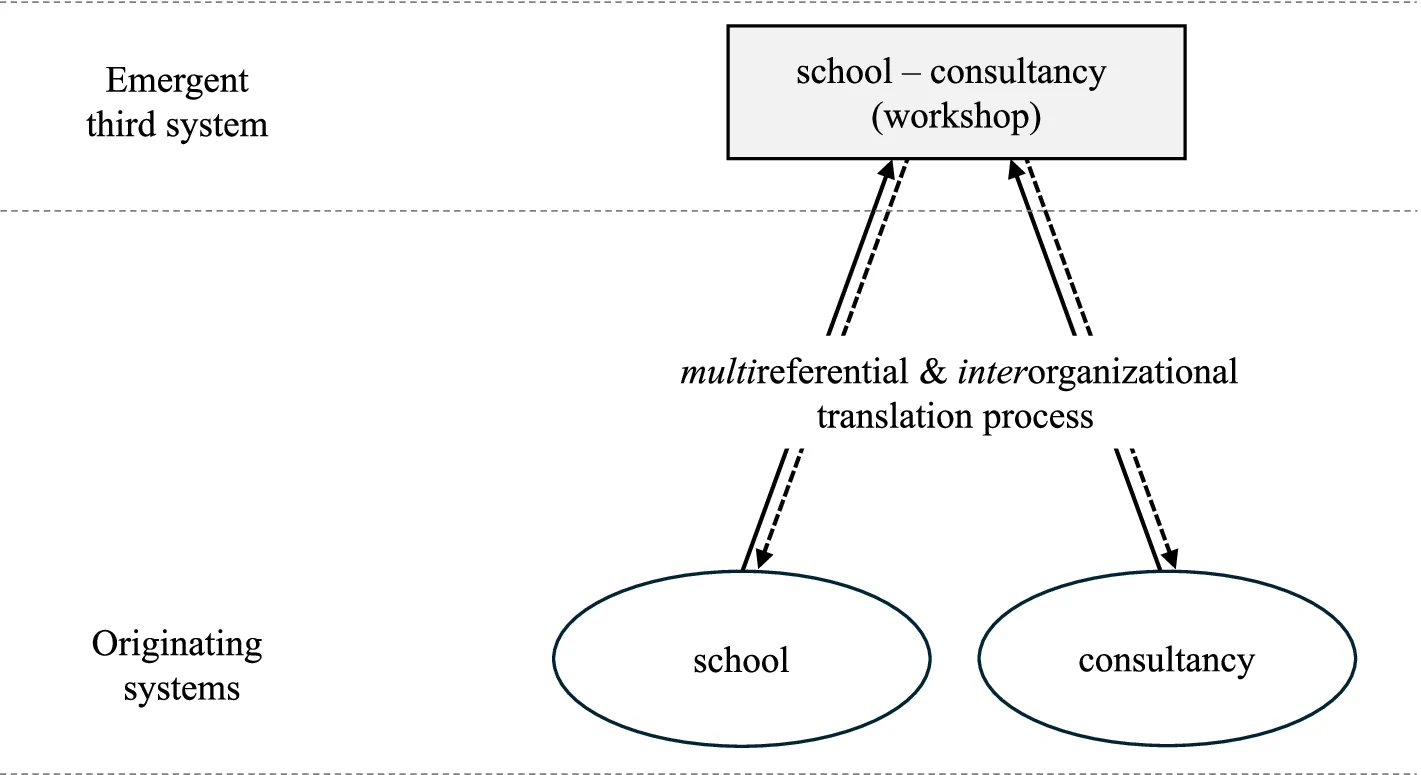
Non-Cartesian intervention in emergent third system.

Assuming that, within the workshop, interorganizational coordination between the primary school and the consulting organization does in fact take place at the operational level—a necessary precondition of the translation process—the findings of the preceding analysis indicate that this requires a distinct system/environment distinction, one that encloses the interorganizational system with multireferential communicative capacity on the left-hand side of the distinction. The emergent formation of such an autonomous system/environment distinction—that is, of a new social system arising from two (or more) organizational systems—presupposes a situation of double contingency. In the course of the implementation process, the organizations enter into a relationship of mutual engagement. The divergence in how each system experiences the event—and any attendant interest in resources (Kron and Dittrich, 2002)— gives rise to imperatives for action: “Double contingency creates pressure to act” (Luhmann, 1995, p. 168). The operationally closed organizations observe one another and achieve mutual adjustment through internal operations, thereby constructing reciprocal expectations. What emerges from this process is neither a combination nor an intermediate formation, but rather a third system (*Drittsystem*)—a system *sui generis*—which in turn constitutes its own system boundary, outside of which the originating systems (school and consulting organization) are perceived as environment (see Figure 3). The third system, therefore, does not contain the originating systems: “They are not elements of the third system” (Fuchs, 1999a, pp. 94–95). Instead, (interorganizational) communicative operation proceeds through recursive decision-making processes in the course of the third system’s autopoiesis. Crucially, the third system (workshop) and the originating systems cannot be understood in terms of the Cartesian dual: third systems are neither “a subject or subjects for subjects, nor an object or objects for objects that are subjects” (Fuchs, 1999a: 95). Communication does not align the perspectives or the operations of the originating systems; rather, it constitutes an *emergent order* that stands over and against those systems. If, for instance, a decision is made within the third system “workshop” to implement new learning methods, the school (and its teachers) is certainly involved and structurally coupled with the third system; yet the operational closure of the autopoietic systems (both the originating systems and the third system) remains intact. The third system “workshop” is confronted with the expectations and programs of the originating systems; however, the successful translation and re-specification of these inputs constitute part of the third system’s own autopoiesis.

Subsequent decisions of the third system thus connect to preceding decisions of the third system. From the perspective of the originating systems, the events in which they are involved are indeed perceived—yet processed in accordance with their own internal logic. Fuchs (1999a, p. 104) points out that the originating systems, which are thus excluded by the emergent third systems, are, paradoxically, reincluded—for it is “clearly about people, organizations, families, when [third] systems open their game”.

Much like the non-Cartesian intervention systems described by Fuchs, the operations of the workshop likewise transform conditions of normality into conditions of non-normality. The extent to which the problem configurations and decisions addressed in this process represent more than mere environmental phenomena for the originating systems—that is, the extent to which the re-entry of the originating systems can be achieved in a sustainable manner—is ultimately a question of addressability (Fuchs, 1997). As noted above, addressability is in principle given for both organizations and the workshop participants alike. But addressability is primarily a projective occurrence, and addresses are “communicative artifacts” (Beckmann, 2024, p. 440). The originating (organizational) systems are thus computed within the third system merely as communicatively existent social addresses (dashed arrow lines in Figure 3). Analogously, people, in and through communication, appear only as “persons”—that is, as “communicatively produced addresses for expectations, attributions, and meaning processors” (Nassehi, 2011, p. 173). Both are attributed with the capacity for self-reference. Where, however, a dislocation of communicative addresses occurs—as when, for instance, classroom instruction substitutes for the workshop setting—polycontextural complications arise accordingly, given that the “reachability” of those attribution points beyond the third system is not guaranteed. This can be observed with particular clarity when the unit of analysis is shifted to the level of individual members.^24^ Even where sustainability can be implemented within the (emergent) third-system communication—that is, as part of the decision premises in the course of multireferential problem specification—translation into the originating systems remains far from guaranteed. Without venturing into the esoteric, one might speak of a “polycontextural consciousness” (Fuchs, 1997; Farzin, 2006) among the participants, in which polycontexturality manifests as a sociality that imprints itself upon action. The inclusion and exclusion of addresses is communicatively produced and subject to social, substantive, temporal (and spatial) ordering. Implementation is blocked here by an *a-addressability of the communicative actors*^25^ and of the third system as such. As a result of this second-order polycontextural complication, re-combination remains confined within the emergent intervention system.^26^

### 3.5 Final generalization

In the final step, we seek to raise the level of generalization once more and to broaden the inquiry into the conditions under which sustainable programming can be implemented within organizations (step 5 in Figure 1). In doing so, we follow our non-subsumptive methodological approach. As argued above, our aim is less a revision of the social-ontological premises of observation in light of the society-theoretical insights gained (as articulated by, e.g., Lindemann, 2025; Fröhlich, 2025) than the announced feedback with respect to the society-theoretical presuppositions.

**Figure 4:**
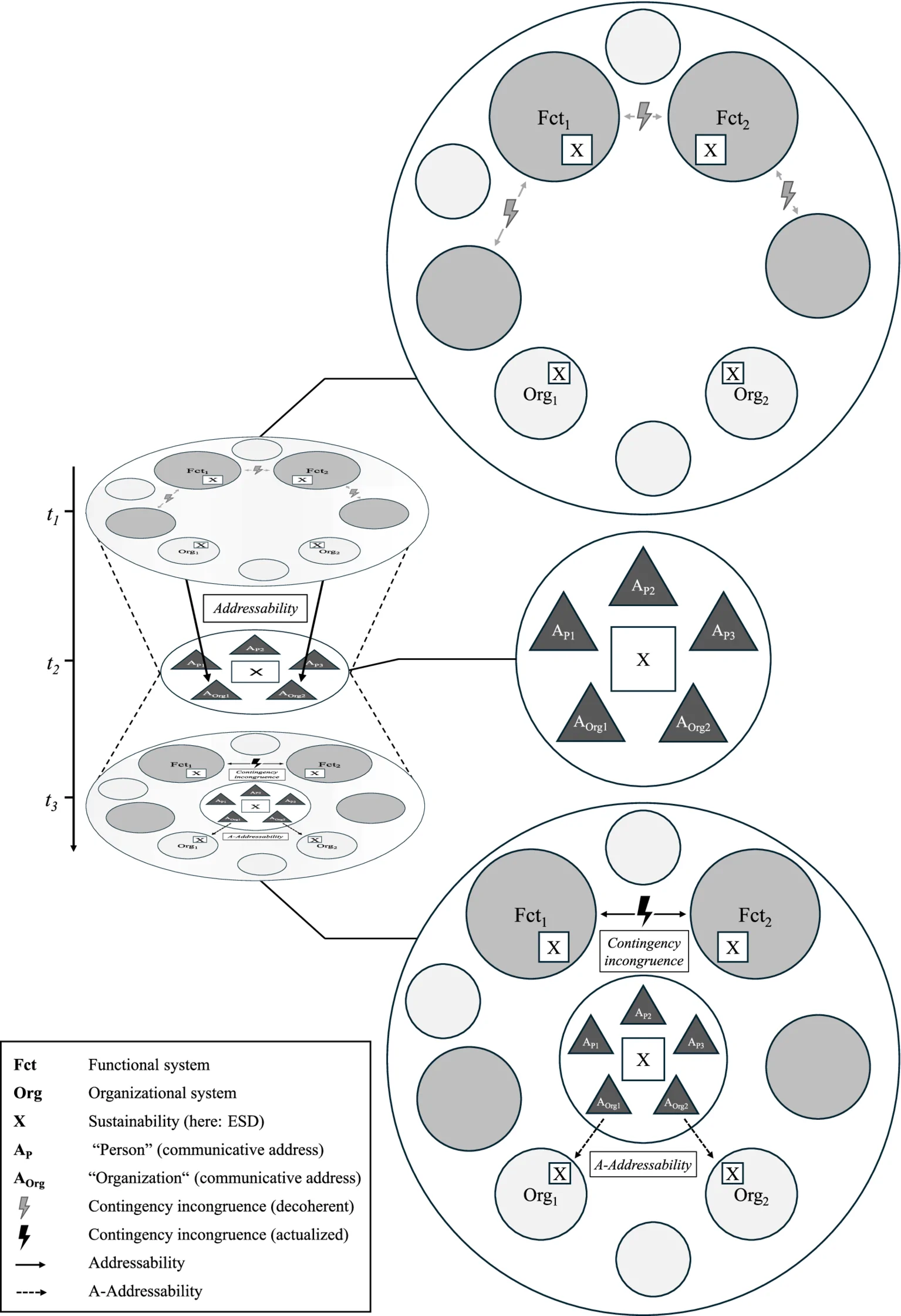
Re-combination and polycontextural complications.

The fundamental problem of integrating multifunctional expectations is addressed organizationally in a variety of ways (Besio and Meyer, 2022). The iterative procedure ultimately led through the needle’s eye of an emergent intervention system. Figure 4 visualizes the reconstructed first- and second-order translation processes as well as the identified polycontextural complications. Corresponding to the highest level of generalization, we accompany the generalization of the intersystemic process with basic mathematical notation, in order to maximize distance from its semantic, case-specific content. Here, X(s) maps sustainability (here: sustainable education) as a function of the system reference s , with $\;s\in M,M=\{{\mathrm{Fct}}_{i};{\mathrm{Org}}_{i};{\mathrm{Int}}_{i}\}$ .

At time $t_{1}$ , the organizational systems ${\mathrm{Org}}_{1}$ and ${\mathrm{Org}}_{2}$ (here: school and consulting organization) are situated in their initial state prior to the implementation or re-combination process. The societal expectations or irritations $X({\mathrm{Fct}}_{i})\;$ are formed monoreferentially within the functional systems ${\mathrm{Fct}}_{i}$ . In the case considered here, both the education system and the political system generate system-specific, monoreferential expectations of sustainable education $X({\mathrm{Fct}}_{i})$ . Equally, the expectation is addressed within the organizations in an undifferentiated manner yet remains constrained as, e.g., X(school) , by their respective established programs. In the primary school at this point, ecological sustainability exists almost exclusively as a learning goal within general studies and biology instruction. An integration of the diverging polycontextural expectations across the systems has not yet taken place.

With the constitution of the (interactional) intervention system as the needle’s eye at time $t_{2}$ , the first- and second-order translation and re-specification processes commence. ${\mathrm{Org}}_{1}$ and ${\mathrm{Org}}_{2}$ , along with the members of these systems, now form communicative addresses $A_{\mathrm{Org}}$ and $A_{P}$ *within* the intervention system. The diverging expectations are re-combined by translating specified concepts (here: “future skills”) into the established logic (primary school) and re-specifying them as $X({\mathrm{Int}}_{i})$ . In the case of the primary schools, this occurs, as outlined above, in the context of the workshop. Through translation practices, the diverging expectations are translated and re-specified to the point where they become manageable as X(lesson) for the school at the level of interaction.

Time $t_{3}$ —situated temporally and spatially beyond the intervention system—represents the blocked re-combination. The translation $X({\mathrm{Int}}_{i})$ , as a communicative event of the emergent third system, is unavailable to the originating system ${\mathrm{Org}}_{1}$ . The communication, together with the communicatively computed “persons,” appears to lie beyond the reach of the organizational system as a consequence of the a-addressability of the operationally closed third system. The contingency incongruence of the diverging logics of translation that emerges at $t_{3}$ beyond the first-order polycontextural complication is *actualized* beyond the third system (second-order polycontextural complication). The ostensibly translatable duality “remains” a conflictual dualism. In the case examined here, this complication accordingly results in an a-addressability of the communicative addresses of the workshop. The ostensibly successful translation and re-specification of ESD X within the workshop does not leave the intervention system. The individual systems continue to operate with their monocontextural conceptions of sustainability. Divergence is actualized; successful re-combination largely fails to materialize.

The relationship between the first- and second-order complications is complex. The assignment of translation processes and emerging blockages is intended to suggest neither a temporal sequence nor a qualitative hierarchy. The contingency incongruence of external and established program logics is rather subject to a process of decoherence (grey and white lightning bolds in Figure 4): it exists prior to the implementation process yet only manifests through its problematization within the framework of the intervention system—the complication simultaneously *exists* and does *not exist* until it is concretized and addressed in the second-order translation process (see $t_{1}$ in Figure 4).

The first-order polycontextural complication (contingency incongruence) is potentially present in every re-combination process yet depends fundamentally on the environment of the organizations—in the present case, primarily on the education system and its coding—and accordingly varies from case to case. The identified second-order polycontextural complication (a-addressability and the emergence of the intervention system), by contrast, points to a more general condition of re-combination processes. We do not attribute a dysfunctional unavailability to every organizational re-combination process, and certainly not to every organizational intervention system. Such an undifferentiated generalization would undermine the self-imposed reflexive mandate of this study (see *Prelude*). A-addressability must, however, be taken into account and empirically examined at least whenever a complex asymmetric translation process is present. Consistently adopting the premise of operational closure at the level of organized social systems (Kneer, 2001) implies that the interorganizational communication often demanded by the translation process can occur exclusively within emergent third systems. The present study demonstrates that the polycontextural configurations and blockages identified become visible only under the premise of operational closure and along diverging horizontal and vertical couplings.

## 4 Discussion and conclusion

Proposed solutions to today’s increasingly turbulent and complex crises frequently hinge on the organizational re-combination of multifunctional rationalities. Systems-theoretical studies of such organizational processes, however, tend to confine themselves to vague organizational self-descriptions—e.g., cases in which linking profit maximization with ecological sustainability represents, above all, a marketing decision. Much like “organized hypocrisy” (Brunsson, 2017), it is thus the “formal façade” rather than the internal re-combination processes that tends to be analyzed. This methodological gap is compounded by a further, more material one: schools in general, and primary schools in particular, remain a comparatively neglected “material research object” (Krause, 2021) within systems-theoretical organizational sociology. A sociological explanation of intersystemic implementation dynamics therefore requires closer and reflexive examination of the concrete events constituting these processes.

Our heuristic systems-theoretical framework pursues two essential objectives. Drawing on the case of ESD in German primary schools, the article has sought to illuminate the implementation process by developing a sensitizing heuristic. In order to disclose, at the meta-theoretical level, the iterative process of theorizing and empirical procedure along the analytical reconstruction of the implementation process, it was necessary to ensure a reflexive—i.e., transparent—engagement with theoretical concepts. The choice of a dogmatic Luhmannian systems theory, which at first glance may appear counterintuitive given its frequent reception as inherently conservative, rests on two central theoretical considerations: (1) Rather than drawing on a flexible theoretical “toolkit” (Foucault, 2014; Reckwitz, 2021) that permits precise explanatory work *with* multiple theories (Anicker and Armbruster, 2024, pp. 2–3), we deliberately employ a largely dogmatic systems-theoretical apparatus. So as to (a) enable theoretical work that is controlled, transparent in its premises, and—precisely for that reason—open to critique. For example, the action-shaping effects of distinct communicative contextures only become visible through a theoretically coherent choice of addressability rather than, for instance, roles. Aspects that thereby fall from view can be clearly defined in terms of the “reflexive reductionism” established at the outset. The society-theoretical perspective adopted here, together with the meta-theoretical heuristic (Figure 1), enables (b) a controlled variation of the level of generalization. At a high level, it enables the articulation of implications of these findings with respect to intersystemic transformation processes (see section 3.5). At a lower level, it affords connectivity to existing research, even as the phenomena are reframed in distinctive terms, as becomes evident across the polycontextural complications identified above.^27^ The reflexivity underlying (a) and (b) renders a concrete feedback effects on the society-theoretical premises of the initial reconstruction (*placement*) traceable in the sense of a non-subsumptive methodological approach. (2) The sharply contoured analytical forms produced by this correspondingly conservative reconstruction serve as a specific “contrast medium” for the analysis of organizational transformation processes, rendering the identified polycontextural complications visible and making them available for explanatory purposes (systems-theoretical heuristic).

In light of the “waning metatheoretical interest [and] a general decline in theoretical discourse” (Anicker et al., 2026, p. 7), this contribution has argued from the outset for a rigorous reflexivity regarding the “theory-ladenness of all observation” (Hirschauer, 2008, p. 167). What emerges from this is the suggestion that systems theory functions not merely as a heuristic interpretive framework but can—as a comprehensive research program—structure the entire research process: from the differentiation-theoretical systematization of the field through the selection and justification of methodological approaches to the theory-guided analysis and (re)embedding of data. In this manner, it affords an integrative perspective that connects theoretical reflection, methodological procedure, and empirical observation, thereby guiding the analysis of social transformation processes in their full complexity. In this sense, systems theory does not offer linear predictions of transformation processes—such as the implementation of ESD under polycontextural conditions: rather, it provides the analytical capacity to render visible, through second-order observation, the “conditions of spaces of possibility” (Vogd, 2007), along diverging couplings and contextures, thereby enabling differentiation between (un)realistic scenarios (Anicker, 2026, p. 235). As the polycrisis intensifies, so too does the demand for intersystemic coordination—and with it, the need for conceptual tools adequate to the complexity of such dynamics. The systems-theoretical perspective proves particularly generative in this regard: its rigorous conceptual architecture not only provides an analytical vocabulary for unpacking such polycontextural constellations, but also affords the capacity to develop new concepts that are precisely tailored to the configurations at hand. In this way, systems theory proves to be not merely a heuristic for observing transformation processes, but a productive resource for conceptual innovation in the face of novel societal challenges.

## Data Availability

The datasets are not publicly available due to confidentiality and ethical restrictions but are available from the corresponding author on reasonable request. Requests to access the datasets should be directed to Simon Schmalen, sschmalen@soziologie.rwth-aachen.de.

## Footnotes

1 We distinguish throughout between *social ontology* (or *social-ontological*) and *theory of society* (or *society-theoretical*), social ontology being used here in the broader sense of the German *Sozialtheorie*, covering both epistemic and ontological basic concepts.

2 The “organization school,” for example, no longer encompasses all processes related to the schooling of young people (i.e., “what is written on the label is included”), but only those that can be observed as decisions connecting to further decisions within the school, including the inclusion and exclusion of specific actors (via membership decisions).

3 Herbert Blumer should therefore not be treated as a foundational reference that reliably protects against a simple subsumption logic. Peetz et al. (2011, p. 211), for instance, emphasize that “the path from theory to empiricism is not a one-way street,” without elaborating on this feedback beyond a single illustrative example (see also Peetz, 2019). That this feedback remains confined to society-theoretical preconceptions follows from the distinction between social ontology and theory of society. While the latter serves as a methodological assumption in empirical enrichment—one whose hypothetical status is made explicit—the former provides the constitutive categories of observation, with the important implication that: “It is just not possible to compare the “raw object”, stripped of all interpretation, with the categories that are used to interpret it” (Anicker, 2026, p. 225).

4 The division into phases is primarily guided by research practice. The gradient of generalization could in principle be resolved at a much finer scale, distinguishing individual cycles of enrichment within each phase (on the relationship between empirical enrichment and theorizing, see also Rachlitz et al., 2024).

5 Luhmann’s claim to universality (Luhmann, 1995, p. 15) should not be mistaken for a claim to totality (Schimank, 2007, p. 147). Our aim is *not* to reconstruct ESD in its totality—for example, by following it across the distinct domains of politics, science, or the economy. Rather, in the sense of a *pragmatic reductionism*, we develop a systematization of the specific polycontextural relations germane to the case at hand. As our focus is on the translation of ESD as a political program into the education system, the analysis centers on the interplay between these two domains, while remaining sensitive to interactions with other contextures.

6 Rasfeld proposes allocating a fixed weekly block of at least four consecutive hours for project work as part of the regular timetable, with other subjects yielding portions of their scheduled time.

7 Ecological communication refers to the reflexive inclusion of ecological threats within the operations of social systems (Luhmann, 1989). In the case of organized social systems, this additionally encompasses recursive networks of decision.

8 Kneer (2001, p. 415) rightly identifies an inconsistency in Luhmann’s systems theory: the idea of a “construction principle” according to which functional systems are composed of organizations each clearly assignable to one functional system sits uneasily with the autopoietic premise of the theory. Following this critique, organizations are understood here as autopoietically closed communication systems within society.

9 In the German federal state of North Rhine-Westphalia, schools are legally entitled to two inset days (*pädagogische Ganztage*) per school year, during which regular classes are set aside for school development activities.

10 https://schule-im-aufbruch.de, accessed April 3, 2026.

11 https://www.bne-portal.de/bne/de/einstieg/bildungsbereiche/whole-institution-approach/whole-institution-approach.html, accessed April 3, 2026.

12 In the course of data collection, additional translation formats (“open school”, peer observation) were documented beyond the workshop, which cannot be addressed in further detail here. The “open school” concept seeks re-combination through an multireferential *intra*organizational structure in which students rotate daily across different “departments.” The largely self-contained departments dispense with joint re-specification. The third translation cascade under study is peer observation as an monoreferential *inter*organizational format. Here, schools grant external actors—such as school leaders, teachers, or pedagogical staff—access to their premises and classroom practices, thereby enabling direct exchange within a shared functional reference. While workshops operate primarily at a conceptual level, peer observation enables direct, practice-oriented engagement with implementation *in situ*.

13 “Empty” categories of the various referentialities in decision communication and re-specification, for instance, form open observation grids or thematic blocks in data collection.

14 https://www.bmbfsfj.bund.de/bmbfsfj/themen/bildung/fruehe-bildung/bildung-fuer-nachhaltige-entwicklung-274304, accessed May 4, 2026.

15 The term “classical learning goals” derives here from the ethnographic data collected during a workshop as well as from the interviews and was defined in opposition to the “future skills.” In contrast to the “future skills” the “classical learning goals” designate the non-competency-oriented learning goals that often form the starting point of the implementation process.

16 The schools studied differ according to their stage of implementation (“classical primary school,” “transformation school,” “ESD school”).

17 In the 2023/2024 school year, ESD is embedded in teaching at around 12 percent of German schools. See the ESD indicator of the Statistical Office: https://dns-indikatoren.de/4-1-c/, accessed May 22, 2026.

18 To be distinguished from this are translations in which organizations *symmetrically* translate two external logics into one another. This is achieved by disregarding potentially conflicting elements and considering only those that are compatible (Besio and Meyer, 2022, pp. 556–557).

19 Fieldnotes and interviews were translated by the authors.

20 CON1 works alongside her roles as a teacher and school leader also as a consultant and is part of the multiprofessional team of the consulting organization. Through such constellations, the consulting organization deliberately draws on “field competence“in order to generate “systemic connectivity,” as explained by a managing director of the organization during an interview.

21 Beyond organizations with correspondingly multireferential purpose-programs—such as sustainable energy cooperatives (Besio and Meyer, 2022)—or startups, in which no established logic exists in the first place, it remains questionable whether a symmetrical translation is possible at all.

22 That all psychic systems—i.e. all students—are non-trivial machines is self-evident and would be disputed by no educator; the point here is rather that the education system educates them *as if* they were trivial machines. This is grounded in the systemic problem of the separation and re-combination of coding and programming: “Trivial machines can be observed for errors and selectively treated on the basis of correct or incorrect operation” (Luhmann, 1987a, p. 194).

23 In accordance with the teacher’s quote cited above, Luhmann states: “But they cannot avoid this reference of their behavior to careers *since careers are a given possibility* and since they are the standard instrument of inclusion by which persons are appointed to positions throughout many systems” (Luhmann, 1989, pp. 101–102, emphasis added).

24 The theoretical decision in favor of the concept of addressability rather than, e.g., role follows from the strictly non-subsumptive methodological approach. The concept of “role” conflates the functions of expectation-bundling and communicative attribution, such that it is ultimately not the communicative context but the reference group that becomes decisive. In the present case, however, the role expectations of, e.g., students (classroom) and those of workshop participants do not necessarily diverge—and yet the addresses do diverge; it is *ergo* communication itself that computes *exclusive* addresses.

25 The hyphenated notation prominent in systems theory here serves—as do the other such constructions we employ—both to defamiliarize and to figure a positive theoretical object. The prefixed “a-” is to be read as an *alpha privativum*; it marks, however, not the mere absence of an address but an autonomous, structurally produced mode of withdrawal from the address form.

26 The problem of a-addressability can be observed analogously in the context of peer observations: “You come back from the visit. How do you then carry that into your team? […] Not just “that looked nice, it was pleasant,” but transporting it to what one actually has” (CON1).

27 While, at the first-order level, existing research has pointed to tensions between, e.g., learning objectives and assessment requirements on the one hand, and holistic, cross-curricular competencies and ESD elements on the other (Budde and Blasse, 2023; Lange et al., 2026), our systems-theoretical reconstruction extends this observation by tracing it back to distinct communicative contingency logics. At the second-order level, while studies have examined, for instance, institutional, infrastructural, and individual obstacles to transferring teacher training into classroom practice (for an overview see Amemasor et al., 2025), here, our perspective foregrounds the action-shaping effects of communicative structures themselves.
